# Latent profiles of digital health literacy and perceived stigma in burn patients: a cross-sectional study

**DOI:** 10.3389/fpubh.2025.1702458

**Published:** 2026-01-06

**Authors:** Fengwen Yue, Liping Liu, Qingjiang Huang, Huanhuan Dai, Lanfang Zhang, Ting Jiang

**Affiliations:** 1 Burn Plastic and Aesthetic Surgery, Beijing Anzhen Nanchong Hospital of Capital Medical University and Nanchong Central Hospital, Nanchong, Sichuan, China; 2 Department of Medical Imaging, Affiliated Hospital of North Sichuan Medical College, Nanchong, China

**Keywords:** appearance anxiety, burn patients, digital health literacy, latent profile analysis, perceived stigma, social support

## Abstract

**Background:**

Prior research has consistently demonstrated that higher levels of digital health literacy contribute positively to improved mental health outcomes and overall quality of life among patients. Nevertheless, the interplay between digital health literacy and the experience of perceived stigma—particularly among burn patients—remains underexplored, and the potential heterogeneity within this relationship has not been adequately addressed.

**Methods:**

This cross-sectional study, conducted from June to July 2025, recruited 534 burn patients (mean age 31.05 ± 9.52 years; 61.0% male) from three tertiary hospitals in Sichuan Province, China. Participants completed validated scales assessing digital health literacy, social support, appearance anxiety, perceived stigma, and demographics. Data were analyzed using Pearson correlations, latent profile analysis (LPA) with fit indices, univariate analyses (chi-square tests and *t*-tests), and multinomial logistic regression. Significance was set at *p* < 0.05.

**Results:**

Digital health literacy was negatively correlated with perceived stigma (*r* = −0.386, *p* < 0.01). LPA identified two profiles: “low digital health literacy-high perceived stigma” (40.2% of sample) and “high digital health literacy-low perceived stigma” (59.8% of sample). Univariate analyses revealed significant differences between profiles in education (χ^2^ = 35.795, *p* < 0.001), social support (*t* = 18.848, *p* < 0.001), and appearance anxiety (*t* = 6.222, *p* < 0.001). Multinomial logistic regression, with high digital health literacy-low perceived stigma as reference, showed social support as a protective factor (OR = 0.125, *p* < 0.001), appearance anxiety as a risk factor (OR = 1.284, *p* = 0.037), and junior high/high school education as a risk (OR = 0.149, *p* = 0.008).

**Conclusion:**

This study confirms heterogeneity in digital health literacy and perceived stigma among burn patients, with social support and appearance anxiety as key influencers. Findings support targeted interventions to enhance digital health literacy and reduce perceived stigma, advancing precision psychological care for burn survivors.

## 1 Introduction

Burn injuries result from exposure to heat, electricity, chemicals, radiation, or other hazardous substances (1, 2). Such injuries lead to localized tissue destruction and trigger a cascade of systemic pathophysiological responses (3). A hallmark consequence of burn injuries is the alteration of physical appearance, which may include the formation of scars, pigmentary changes, facial disfigurement, hair loss, impairment of dynamic facial expressions, and damage to the skin of the extremities (4, 5). These physical changes are often accompanied by significant functional impairments and give rise to profound psychological distress. In particular, alterations in appearance can provoke a sense of estrangement from one’s former self-image, disrupting the individual’s ongoing cognitive perception of personal identity and often precipitating intense feelings of self-rejection and shame (6–8). More critically, burn patients are frequently subjected to social stigma, leading to interpersonal alienation or exclusion (9). Such social marginalization further intensifies emotions of loneliness and rejection, ultimately compromising the integrity of their social support systems. Recent years have witnessed a growing body of research aimed at alleviating the perceived stigma among burn patients through diverse intervention strategies, including mindfulness-based approaches, peer support programs, and interventions targeting psychological flexibility (10, 11). Despite these advances, the relationship between digital health literacy and perceived stigma in burn patients has received comparatively little empirical attention.

Digital health literacy is broadly defined as an individual’s capacity to seek, comprehend, evaluate, and apply health-related information within digital environments (12). Prior investigations have indicated that exposure to unverified or low-quality health information may adversely affect patients’ perceptions of stigma (13). Specifically, contradictory or misleading medical content can engender confusion in treatment decision-making, erode trust in healthcare professionals, and heighten feelings of social alienation (14). Moreover, excessive consumption of negatively valenced health-related content can lead to algorithmic reinforcement of similar distressing material. Consequently, fostering digital health literacy among burn patients is of paramount importance. Individuals with higher levels of digital health literacy are better equipped to access reputable online sources offering evidence-based rehabilitation strategies, psychological support services, and connections to patient communities (15, 16). This enhanced access facilitates a more accurate understanding of disease prognosis and contributes to the reduction of self-imposed stigma stemming from physical disfigurement or functional limitations.

Despite growing recognition of the importance of health literacy, many prior cross-sectional studies have primarily examined the broad relationship between general health literacy and perceived stigma (17, 18), often yielding mixed results. For example, Kurki, Gilbert (19) reported indirect evidence suggesting that health literacy does not exert a significant influence on perceived stigma among individuals with mental illness, as inferred through patterns of digital over-engagement. In contrast, Yang, Hu (20) identified that greater health literacy is associated with reduced perceived stigma related to help-seeking behaviors. Similarly, Crowe, Mullen (21) affirmed a significant negative association between perceived stigma and health literacy, corroborating the findings of Yang, Hu (20). These discrepancies may stem from methodological limitations inherent in traditional variable-centered research approaches, which often fail to account for variability across different patient populations, contextual factors, and specific variables under investigation (22). Of particular note, prior studies have largely neglected to explore the nuanced relationship and heterogeneity between digital health literacy and perceived stigma specifically within the burn patient population, and even fewer have systematically examined the factors that may influence this relationship.

According to social cognitive theory (23), social support is recognized as a fundamental determinant influencing patients’ acquisition of digital health knowledge and their perceptions of discrimination (24, 25). Notably, the availability and quality of social support may vary considerably across different subgroups of burn patients. Robust social support, encompassing emotional encouragement, informational guidance, and tangible assistance from family members, peers, or healthcare providers (26, 27), can bolster patients’ confidence and competence in navigating digital health resources. This, in turn, enhances their ability to obtain credible medical information and engage in effective self-management of their condition (28, 29). In contrast, a paucity of social support may intensify feelings of isolation, restrict engagement with digital health platforms, and impede the development of health-related knowledge (30, 31). Accordingly, we hypothesize that the degree of social support experienced by burn patients may significantly shape the latent profiles of digital health literacy and perceived stigma. Specifically, higher levels of social support may mitigate perceptions of discrimination by promoting self-acceptance and reducing self-consciousness related to bodily appearance or social interactions. Conversely, individuals with limited social support may be more susceptible to internalizing negative societal attitudes, thereby exacerbating experiences of discrimination.

According to social comparison theory, we further posit that appearance anxiety constitutes another critical factor influencing the digital health literacy and perceived stigma of burn patients. For burn patients, such comparisons can intensify feelings of appearance anxiety, which subsequently interferes with social interactions and fosters heightened concern about being judged by others, which contribute to the experience of perceived stigma (32). Additionally, appearance anxiety may motivate individuals to actively seek out medical and health-related information pertaining to appearance restoration (33). However, the behavioral outcomes of such information-seeking are largely contingent upon how individuals access and interpret available information (34). Thus, appearance anxiety is intricately linked to both digital health literacy and the experience of perceived stigma among burn patients.

In light of the identified research gaps, this cross-sectional study aims to: (1) examine the relationship between digital health literacy and perceived stigma in burn patients; (2) use latent profile analysis to identify heterogeneous subgroups based on digital health literacy and stigma, including associated demographics; and (3) investigate the role of social support and appearance anxiety as influential factors within these potential categories. This study provides clinically relevant insights for healthcare providers and family members aiming to develop targeted interventions to reduce self-stigma and enhance the overall well-being of burn patients.

## 2 Method

This cross-sectional study adhered to the STROBE guidelines for reporting observational research (35). All procedures were conducted in accordance with ethical standards, and data were analyzed using validated statistical methods to ensure robustness and reproducibility.

### 2.1 Participants

This study was designed to investigate the relationship and heterogeneity between digital health literacy and perceived stigma among burn patients. Participants were recruited from the Burn and Plastic Surgery Departments of three tertiary A-level hospitals in Sichuan Province, China, between June and July 2025. Convenience sampling was employed, whereby all eligible patients admitted as inpatients or attending outpatient clinics during the recruitment period were sequentially approached by trained research assistants until the target sample size was achieved.

#### 2.1.1 Data collection process

The research team initiated contact with the director of the hospital’s Burn and Plastic Surgery Department to explain the objectives and scope of the study. Questionnaires were developed based on validated instruments and administered in a mixed-mode format: primarily electronically via the Credamo platform^1^, a professional online data collection service widely used in academic research in China. Credamo features a large participant pool (over 3 million registered users, primarily university students and diverse demographics), supports rapid survey deployment (e.g., collecting thousands of responses quickly), and includes built-in quality controls such as attention checks, anti-fraud algorithms to detect patterned or inattentive responses, and real-time data monitoring to ensure validity and reliability. It also offers secure data handling compliant with ethical standards, with responses anonymized and exported via encrypted channels. For participants preferring or requiring it (e.g., due to limited digital access or familiarity), paper-based versions were provided on-site. Trained research assistants facilitated the process: they screened for eligibility, explained the study verbally and in writing (including purpose, risks, benefits, confidentiality, and voluntary nature), and obtained written informed consent. Electronic questionnaires were completed on hospital-provided tablets or participants’ devices, with assistants available for neutral guidance (e.g., clarifying item wording without suggesting answers). Paper versions were administered similarly, with responses later digitized into Credamo for unified analysis. Each questionnaire takes about 5–6 min to complete. Participants received both verbal and written explanations of the study purpose, potential risks and benefits, and confidentiality protection before providing written informed consent. Ethical approval, including consent procedures, was granted by the Academic Ethics Committee of Nanchong Central Hospital (Approval No.: 2025116). To minimize bias, responses were anonymized, and participants were assured of voluntary withdrawal without repercussions.

#### 2.1.2 Inclusion and exclusion criteria

Inclusion criteria were as follows: (1) participants must not have any significant mental disorders or speech impairments; (2) participants must have a clinical diagnosis of burns confirmed by a qualified burn specialist; (3) participants must not have taken part in any similar research studies within the past week; and (4) participants must have provided signed informed consent.

Exclusion criteria included: (1) individuals diagnosed with severe mental illness or those lacking normal speech capabilities; (2) participants who had been involved in any cross-sectional study during the previous week; (3) individuals whose native language is not Chinese and who experienced difficulty in comprehending the questionnaire content; (4) questionnaires exhibiting highly consistent or patterned responses that suggested inattentive or non-genuine completion; and (5) participants who refused to provide informed consent or who withdrew from the study before completion.

#### 2.1.3 Sample size and demographic information

The target sample size was determined *a priori* based on simulation studies for latent profile analysis, recommending at least 500 participants for accurate profile identification and model stability (36). A total of 550 questionnaires were distributed for data collection. During the subsequent data cleaning process, 4 paper questionnaires were excluded due to physical damage, 9 participants withdrew from the study before completion, and 3 questionnaires were invalidated because of excessively uniform responses, which raised concerns about response validity. The final analysis was conducted on a valid sample of 534 participants, yielding an effective response rate of 97.09%. The sample comprised 326 male participants (61.0%) and 208 female participants (39.0%). In terms of educational background, 42 participants (7.9%) had received primary school education or lower, 213 participants (39.9%) had completed junior high to high school education, and 279 participants (52.2%) had attained undergraduate education or higher. The mean age of the participants was 31.05 years, with a standard deviation of 9.519. Detailed demographic characteristics of the sample are presented in Table 1.

**Table 1:** Summary table of demographic information.

Variable	Items	Number	Proportion
Gender	Male	326	61.0%
	Female	208	39.0%
Education background	Primary school and below	42	7.9%
	Middle school to high school	213	39.9%
	Bachelor degree or above	279	52.2%
Marital status	Married	259	48.5%
	Unmarried	187	35.0%
	Divorced	72	13.5%
	Widowed	16	3.0%
Status of occupation	Worker	275	51.5%
	Unemployment	81	15.2%
	Retirement	6	1.1%
	Student	37	6.9%
	Freelance	135	25.3%
Children	Yes.	278	52.1%
	No.	256	47.9%
Place of residence	City	327	61.2%
	Suburb	207	38.8%
Monthly income level	Less than 1,000 yuan	49	9.2%
	1,001–3,000 yuan	127	23.8%
	3,001–5,000 yuan	193	36.1%
	5,001–8,000 yuan	115	21.5%
	≥8,001 yuan	50	9.4%
Cause of burn	Electric burn	139	26.0%
	Chemical burn	29	5.4%
	Flame	212	39.7%
	Hot substance	154	28.8%
Degree of burn	Mild burns	256	47.9%
	Moderate burns	162	30.3%
	Severe burns	97	18.2%
	Extremely severe burns	19	3.6%
Burn staging	Period of infection	214	40.1%
	Acute fluid exudation	129	24.2%
	Period of repair	191	35.8%
Burn surgery	Yes.	257	48.1%
	No.	277	51.9%
Age		31.05 ± 9.519	

### 2.2 Measurement tools

#### 2.2.1 Digital health literacy scale

In this study, the Digital Health Literacy Scale developed by van der Vaart and Drossaert (37) was employed to assess the digital health literacy levels of burn patients. The scale comprises 21 items distributed across seven dimensions: operational skills, information searching, reliability evaluation, determining relevance, navigation skills, adding self-generated content, and protecting privacy. For its application in the Chinese context, the scale was translated into a Chinese version by Xie, Hu (38), who also validated its cultural adaptability among the Chinese older adults population. Responses were recorded using a 7-point Likert scale, where 1 indicates “strongly disagree” and 7 indicates “strongly agree.” Higher total scores reflect greater levels of digital health literacy. Model fit of the scale was evaluated using AMOS 29.0 software, yielding satisfactory results: χ^2^/df = 3.152, TLI = 0.914, GFI = 0.924, AGFI = 0.857, RMSEA = 0.064, and CFI = 0.886. The internal consistency of the scale was also confirmed to be strong, with a Cronbach’s alpha coefficient of 0.935 when administered to burn patients.

#### 2.2.2 Social support scale

To evaluate the level of social support perceived by burn patients, the Perceived Social Support Scale developed by Zimet, Dahlem (39) was utilized. This scale consists of 12 items across three dimensions: support from family, support from friends, and support from other significant individuals. The Chinese version of the scale was adapted and validated for cultural appropriateness by Yang, Xue (40). Responses were measured on a 7-point Likert scale ranging from 1 (strongly disagree) to 7 (strongly agree), with higher scores indicating greater perceived social support. Model fit analysis conducted using AMOS 29.0 software indicated a good fit of the model to the data: χ^2^/df = 2.755, TLI = 0.955, GFI = 0.956, AGFI = 0.963, RMSEA = 0.057, and CFI = 0.963. The scale also demonstrated good internal consistency, with a Cronbach’s alpha of 0.905.

#### 2.2.3 Appearance anxiety scale

The Social Appearance Anxiety Scale developed by Hart, Flora (41) was used to measure the appearance anxiety of burn patients. This scale includes 16 items and has been extensively applied in research involving the Chinese population, thereby confirming its cultural adaptability (42, 43). Responses were collected using a 7-point Likert scale, where 1 represents “strongly disagree” and 7 represents “strongly agree.” A higher total score reflects greater appearance anxiety. Model fit analysis performed with AMOS 29.0 software showed acceptable model fit: χ^2^/df = 3.026, TLI = 0.945, GFI = 0.927, AGFI = 0.905, RMSEA = 0.062, and CFI = 0.952. The internal consistency of the scale was also strong, with a Cronbach’s alpha coefficient of 0.937.

#### 2.2.4 Perceived stigma scale

The Perceived Stigma Scale, originally developed by Reinius, Wettergren (44), was used to assess the level of perceived stigma among burn patients. This scale consists of 12 items grouped into four dimensions: Personalized stigma, Disclosure concerns, Concerns about public attitudes, and Negative self-image. Although initially designed for use with HIV patients, it has since been applied to various other patient populations, including COVID-19 survivors (45) and individuals with inflammatory bowel disease (46). Furthermore, its cultural applicability has been confirmed among Chinese populations in several prior studies (Zhao et al., 2024). Responses were recorded on a 7-point Likert scale (1 = strongly disagree, 7 = strongly agree), with higher scores indicating greater levels of perceived stigma. Model fit analysis using AMOS 29.0 software indicated a good fit: χ^2^/df = 3.575, TLI = 0.946, GFI = 0.941, AGFI = 0.915, RMSEA = 0.070, and CFI = 0.955. The scale also exhibited good reliability, with a Cronbach’s alpha of 0.920.

### 2.3 Data analysis

Data were analyzed in stages to ensure reproducibility. First, AMOS 29.0 assessed scale model fits. SPSS 27.0 conducted reliability testing (Cronbach’s *α*), common method bias (Harman’s single-factor test), descriptive statistics, and Pearson correlations. Mplus 8.0 performed LPA on digital health literacy and perceived stigma scores, evaluating models with 1–5 profiles using AIC, BIC, aBIC (lower values indicate better fit), Entropy (>0.8 for classification accuracy), Lo–Mendell–Rubin (LMR) test, and Bootstrapped Likelihood Ratio Test (BLRT; *p* < 0.05 indicates better fit over k-1 model).

Univariate analyses compared profiles using chi-square tests for categorical variables and independent t-tests for continuous variables (e.g., age, social support, appearance anxiety). Significance was set at p < 0.05 (two-tailed).

Finally, multinomial logistic regression in SPSS 27.0 identified predictors of profile membership, with the “high digital health literacy-low perceived stigma” profile as the reference category. All variables significant in univariate analyses were included as independent predictors to control for confounding: education level (categorical: primary/lower as reference), marital status (categorical), employment status (categorical), place of residence (categorical: urban/rural), monthly income (categorical), burn severity (categorical), social support (continuous score), and appearance anxiety (continuous score). Odds ratios (OR) with 95% confidence intervals (CI) were reported. To assess potential multicollinearity among predictors, variance inflation factors (VIF) were calculated for continuous variables (social support and appearance anxiety) after dummy-coding categorical predictors, with VIF < 5 indicating acceptable levels (consistent with standard thresholds for logistic regression).

Although the bivariate correlation between digital health literacy and social support was relatively high (r = 0.824), this did not result in multicollinearity in the regression models. This is because digital health literacy was not entered as an independent predictor in the multinomial logistic regression—it was incorporated into the outcome variable through the latent profile classification. The VIF values were calculated only among the actual independent predictors (e.g., social support and appearance anxiety) included in the model. As a result, the low VIF values accurately reflect the absence of multicollinearity among the predictors used in the regression analysis.

Missing data were minimal (<1% across variables, primarily due to exclusions during cleaning) and handled via listwise deletion to maintain data integrity without imputation, given the low rate and cross-sectional design. To address potential biases, anonymity was ensured, item sequencing randomized where possible, and Harman’s test confirmed no substantial common method bias (first factor: 27.929% variance). All analyses used default software settings unless specified, with *p* < 0.05 for significance.

## 3 Results

### 3.1 Common method bias test

Since this study relied exclusively on self-reported measures and utilized a uniform scale format across all variables, it was inherently susceptible to generating spurious correlations or obscuring genuine relationships (47). Such methodological limitations can significantly compromise the validity of the findings. Furthermore, burns patients are particularly prone to responding in ways they perceive as socially desirable (48), which may further diminish the authenticity and accuracy of their reported data.

To mitigate these potential biases, several procedural safeguards were implemented. During the research implementation phase, participants were explicitly assured that their responses would remain strictly confidential and would be anonymized, thereby minimizing concerns related to social desirability. Additionally, during the questionnaire development stage, careful attention was given to the sequencing of items to prevent the introduction of any unintended logical cues or response patterns.

To empirically assess the presence of common method bias, all measurement items were subjected to an exploratory factor analysis incorporating Harman’s single-factor test. The analysis revealed that the variance explained by the first extracted factor was 27.929%, which remained well below the commonly accepted threshold of 40%. This finding provides strong evidence that common method bias was not a substantive issue in the current study.

### 3.2 Descriptive statistics and correlation analysis

This study conducted comprehensive descriptive statistical analyses and correlation assessments for all core variables—including digital health literacy, social support, appearance anxiety, and perceived stigma—as well as relevant demographic variables.

As presented in Table 2, the descriptive statistics indicated that the mean scores for all core variables ranged from 4.152 to 4.693, reflecting a generally moderate-to-high level of digital health literacy, social support, appearance anxiety, and perceived stigma among the participants. The standard deviations ranged from 1.060 to 1.218, suggesting a moderate degree of variability across the sample. Regarding the distributional properties, the absolute values of skewness were all below 0.25, and those of kurtosis were under 0.273, indicating that the data for each variable approximated a normal distribution. These characteristics support the appropriateness of employing parametric statistical methods in subsequent analyses.

**Table 2:** Descriptive statistics for core study variables.

Variables	*M*	SD	Skewness	Kurtosis
Digital health literacy	4.395	1.060	−0.129	0.273
Social support	4.693	1.084	−0.250	0.262
Appearance anxiety	4.403	1.177	−0.087	−0.128
Perceived stigma	4.152	1.218	−0.040	−0.047

M = mean; SD = standard deviation.

The correlation analysis results, detailed in Table 3, demonstrated a very strong positive association between digital health literacy and social support (*r* = 0.824, *p* < 0.01), indicating that individuals with higher digital health literacy tended to report greater levels of social support. In contrast, digital health literacy was significantly and negatively correlated with both appearance anxiety (*r* = −0.383, *p* < 0.01) and perceived stigma (*r* = −0.386, *p* < 0.01), suggesting that greater digital health literacy may be associated with reduced psychological burden. Similarly, social support was significantly negatively correlated with appearance anxiety (*r* = −0.297, *p* < 0.01) and perceived stigma (*r* = −0.306, *p* < 0.01), further underscoring its protective role against psychological distress. Appearance anxiety and perceived stigma were positively correlated (*r* = 0.115, *p* < 0.01), indicating that these two constructs may jointly reflect aspects of patients’ overall psychological distress.

**Table 3:** Correlation analysis of study variables.

Variables	1	2	3	4
Digital health literacy	1			
Social support	0.824**	1		
Appearance anxiety	−0.383**	−0.297**	1	
Perceived stigma	−0.386**	−0.306**	0.115**	1

**p < 0.01; *p < 0.05.

### 3.3 Latent profile analysis

To examine the heterogeneity in digital health literacy and perceived stigma among burn patients, LPA was conducted. Models with 1 to 5 latent profile were sequentially fitted, as summarized in Table 4. As the number of latent profile increased, the values of the AIC, BIC, and aBIC continued to decrease, although the rate of decline attenuated after three or more classes. The Entropy value for the 2-profile model was 0.937, substantially exceeding the commonly recommended threshold of 0.8, which indicates excellent classification accuracy. More importantly, both the LMR (*p* = 0.002) and BLRT (*p* < 0.001) for the 2-profile model reached statistical significance, demonstrating that this model provided a significantly better fit compared to the 1-profile model. However, when the number of profiles was increased to 3, the LMR test was no longer significant (*p* = 0.195), and the proportion of the smallest profile dropped to 12.0%, raising concerns about potential over-extraction and instability of the classification. Moreover, the LMR test for the 3-profiles model was not significant, indicating that the additional class did not yield a meaningful improvement over the 2-profiles solution. Transitioning directly from 2 to 4 profiles lacked incremental statistical justification. Therefore, based on a combination of statistical indicators, model parsimony, and interpretability, the 2-profile model was selected as the optimal solution, offering a robust fit while maintaining clear classification boundaries and theoretical relevance.

**Table 4:** Potential profile analysis model fit indicators for digital health literacy and perceived stigma.

Profile	AIC	BIC	aBIC	Entropy	LMR (P)	BLRT (P)	Smallest proportion per class
1	67329.594	67612.100	67402.595				
2	63890.524	64318.564	64001.132	0.937	0.002	<0.001	0.598/0.402
3	62581.947	63155.520	65730.162	0.951	0.195	<0.001	0.120/0.580/0.300
4	61422.495	62141.601	61608.316	0.955	0.042	<0.001	0.459/0.130/0.277/0.134
5	60992.434	61857.074	61215.862	0.951	0.1867	<0.001	0.128/0.448/0.147/0.150/0.127

AIC = Akaike Information Criterion; BIC = Bayesian Information Criterion; aBIC = adjusted BIC; Entropy indicates classification accuracy (values closer to 1 indicate better accuracy). LMR = Lo–Mendell–Rubin test; BLRT = Bootstrapped Likelihood Ratio Test.

### 3.4 Subgroup classification

**Figure 1:**
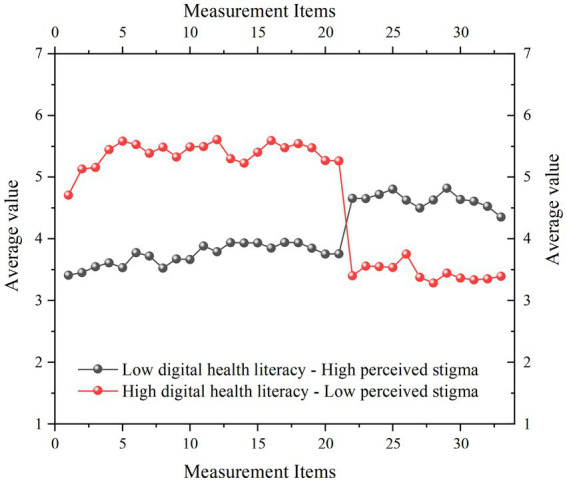
Profile plots of 2 latent categories of digital health literacy and perceived stigma among burn patients. 1–21 are measurement questions for digital health literacy, and 22–33 are measurement questions for perceived stigma.

Based on the optimal 2-profile latent profile model, the standardized score profiles of the two identified subgroups with respect to digital health literacy and perceived stigma are illustrated in Figure 1.

Low digital health literacy–High perceived stigma subgroup: Individuals in this subgroup exhibited lower levels of digital health literacy and higher levels of perceived stigma. This suggests that this group faces greater challenges in accessing, understanding, and utilizing digital health information, while also perceiving higher levels of social discrimination and internalized stigma.

High digital health literacy–Low perceived stigma subgroup: Participants in this subgroup demonstrated higher digital health literacy and lower perceived stigma. This group is more adept at leveraging digital resources for health management and reports lower levels of shame and social exclusion related to their burn injuries.

These two subgroups exhibited pronounced differences in digital health literacy and perceived stigma, highlighting significant heterogeneity within the burn patient population. The High digital health literacy–Low perceived stigma subgroup is indicative of better psychosocial adjustment, characterized by stronger digital health competencies and a reduced psychological burden.

### 3.5 Univariate analysis of demographic information, social support, appearance anxiety, and latent categories

To investigate potential differences in the distribution of demographic and clinical characteristics between the two latent categories (Low Digital Health Literacy–High Perceived Stigma vs. High Digital Health Literacy–Low Perceived Stigma), univariate analyses were conducted. Categorical variables were analyzed using the chi-square test (χ^2^), while continuous variables (including age, social support, and appearance anxiety) were examined using independent samples t-tests. The detailed results are presented in Table 5.

**Table 5:** Univariate analysis of demographic and clinical characteristics according to latent categories of patients.

Variables	Items	Low digital health literacy—High perceived stigma (*N* = 318)	High digital health literacy—Low perceived stigma (*N* = 216)	χ^2^ /*t*	*p*
Gender	Male	193	133	0.042	0.837
	Female	125	83		
Education background	Primary school and below	37	5	35.795	<0.001
	Middle school to high school	146	67		
	Bachelor degree or above	135	144		
Marital status	Married	146	113	19.785	<0.001
	Unmarried	101	86		
	Divorced	58	14		
	Widowed	13	3		
Status of work	Worker	144	131	19.908	<0.001
	Unemployment	63	18		
	Retirement	5	1		
	Student	20	17		
	Freelance	86	49		
Children	Yes.	163	115	0.203	0.653
	No.	155	101		
Place of residence	City	177	150	10.296	0.001
	Suburb	141	66		
Monthly income level	≤1,000 yuan	34	15	10.746	0.030
	1,001–3,000 yuan	81	46		
	3,001–5,000 yuan	119	74		
	5,001–8,000 yuan	63	52		
	≥8,001 yuan	21	29		
Cause of burn	Electric burn	85	54	5.42	0.144
	Chemical burn	18	11		
	Flame	135	77		
	Hot substance	80	74		
Degree of burn	Mild burns	134	122	15.12	0.002
	Moderate burns	100	62		
	Severe burns	68	29		
	Extremely severe burns	16	3		
Burn staging	Period of infection	143	71	8.164	0.017
	Acute fluid exudation	73	56		
	Period of repair	102	89		
Burn surgery	Yes.	169	108	0.51	0.475
	No.	149	108		
Age (M ± SD)		31.66 ± 10.683	30.16 ± 7.418	1.791	0.074
Social support (M ± SD)		4.127 ± 0.881	5.524 ± 0.776	18.848	<0.001
Appearance anxiety (M ± SD)		4.655 ± 1.048	4.031 ± 1.257	6.222	<0.001

Values are χ^2^ for categorical variables and independent-samples *t* values for continuous variables. *p* values represent the level of significance. M = mean; SD = standard deviation.

The univariate analysis revealed no statistically significant differences between the two groups in terms of gender (χ^2^ = 0.042, *p* = 0.837), age (t = 3.206, *p* = 0.074), having children (χ^2^ = 0.203, *p* = 0.653), cause of burn (χ^2^ = 5.42, *p* = 0.144), or history of burn surgery (*t* = 0.51, *p* = 0.475).

However, significant differences were observed across several demographic variables, including education level (χ^2^ = 35.795, *p* < 0.001), marital status (χ^2^ = 19.785, *p* < 0.001), employment status (χ^2^ = 19.908, *p* < 0.001), place of residence (χ^2^ = 10.296, *p* = 0.001), monthly income (χ^2^ = 10.746, *p* = 0.030), and burn severity (χ^2^ = 15.12, *p* = 0.002).

Moreover, the two groups differed significantly in social support (*t* = 18.848, *p* < 0.001) and appearance anxiety (*t* = 6.222, *p* < 0.001). The High Health Literacy–Low Perceived Stigma Group had significantly higher social support scores and significantly lower appearance anxiety scores compared to the other subgroup.

### 3.6 Multivariate logistic regression analysis of latent categories and influencing factors

To identify the key factors associated with patients’ membership in the different latent categories (Low digital health literacy–High perceived stigma vs. High digital health literacy–Low perceived stigma), an ordinal multinomial logistic regression analysis was performed based on the results of the univariate analysis. The latent category served as the dependent variable, with the High digital health literacy–Low perceived stigma group treated as the reference category. All variables that had shown statistical significance in the univariate analysis were included in the multivariate model to control for confounding effects and to isolate independent predictors. The detailed regression results are presented in Table 6. Prior to regression, multicollinearity was assessed via variance inflation factors (VIF) for the continuous predictors (social support and appearance anxiety), after incorporating dummy-coded categorical variables. VIF values were low (social support: VIF = 1.32; appearance anxiety: VIF = 1.19), well below the threshold of 5, indicating no substantial multicollinearity among predictors.

**Table 6:** Ordinal multinomial logistic regression analysis of factors influencing latent categories of burn patients.

Variables	Items	Regression coefficient	Standard error	Wald χ^2^	*P*	OR	LLCI	ULCI
Social support		−2.083	0.196	113.461	<0.001	0.125	0.085	0.183
Appearance anxiety		0.250	0.119	4.37	0.037	1.284	1.016	1.622
Education background	Primary school and below	−1.109	0.709	2.442	0.118	0.33	0.082	1.325
	Middle school to high school	−1.906	0.716	7.087	0.008	0.149	0.037	0.605
	Bachelor degree or above (reference)							
Marital status	Married	0.587	0.452	1.689	0.194	1.799	0.742	4.364
	Unmarried	−0.289	0.876	0.109	0.741	0.749	0.134	4.17
	Divorced	−0.256	0.304	0.708	0.400	0.775	0.427	1.404
	Widowed (reference)							
Status of work	Worker	−0.555	0.33	2.839	0.092	0.574	0.301	1.095
	Unemployment	−0.527	0.469	1.263	0.261	0.59	0.235	1.48
	Retirement	−0.905	1.44	0.395	0.530	0.405	0.024	6.804
	Student	−0.617	0.576	1.146	0.284	0.54	0.174	1.669
	Freelance (reference)							
Place of residence	Yes.	0.034	0.296	0.013	0.909	1.034	0.58	1.846
	No. (reference)							
Monthly income level	≤1,000 yuan	−0.425	0.581	0.534	0.465	0.654	0.209	2.044
	1,001–3,000 yuan	0.001	0.501	0.000	0.999	1.001	0.375	2.673
	3,001–5,000 yuan	−0.242	0.424	0.324	0.569	0.785	0.342	1.803
	5,001–8,000 yuan	−0.108	0.355	0.093	0.760	0.897	0.447	1.799
	≥8,001 yuan (reference)							
Degree of burn	Mild burns	−0.296	0.422	0.492	0.483	0.744	0.325	1.702
	Moderate burns	1.268	0.978	1.680	0.195	3.553	0.523	24.155
	Severe burns	0.02	0.405	0.002	0.961	1.02	0.461	2.257
	Extremely severe burns (reference)							
Burn staging	Period of infection	0.062	0.329	0.035	0.852	1.063	0.558	2.026
	Acute fluid exudation	0.475	0.338	1.981	0.159	1.608	0.83	3.118
	Period of repair (reference)							

OR = odds ratio; CI = confidence interval; LLCI = lower limit CI; ULCI = upper limit CI. *P-*values represent the level of significance.

The overall regression model was statistically significant (χ^2^ = 317.960, *p* < 0.001), indicating that the included independent variables collectively predicted patients’ latent category membership with good accuracy. The results of the analysis revealed the following key findings: Social support emerged as a robust protective factor for membership in the more favorable High digital health literacy–Low perceived stigma category (*β* = −2.083, Wald χ^2^ = 113.461, *p* < 0.001). The odds ratio (OR) was 0.125, indicating that for each one-unit increase in social support, the likelihood of belonging to the “High digital health literacy–Low perceived stigma” group decreased by 87.5%. Conversely, lower levels of social support were associated with a higher probability of being in the less favorable Low digital health literacy–High perceived stigma.

Appearance anxiety was identified as an independent risk factor for membership in the less favorable category (*β* = 0.250, Wald χ^2^ = 4.370, *p* = 0.037). The OR was 1.284, meaning that for each one-unit increase in appearance anxiety, the likelihood of being in the High digital health literacy–Low perceived stigma group decreased—indicating that higher appearance anxiety was associated with a greater likelihood of being in the Low digital health literacy–High perceived stigma group. In other words, elevated appearance anxiety significantly reduced the probability of patients being categorized into the more adaptive subgroup.

Education level also emerged as a significant predictor. Compared to individuals with a bachelor’s degree or higher, those with junior high to high school education levels had a significantly lower likelihood of belonging to the High digital health literacy–Low perceived stigma group (OR = 0.149, *p* = 0.008), reflecting an 85.1% reduction in likelihood. A similar negative trend was observed for individuals with primary school education or below, although this effect did not reach statistical significance (*p* = 0.118).

Within the multivariate model, variables such as burn severity, marital status, employment status, and income level did not demonstrate independent statistical significance (*p* > 0.05). This suggests that their potential influence on latent category membership may be mediated or overshadowed by more proximal psychosocial factors, particularly social support and appearance anxiety.

## 4 Discussion

### 4.1 Summary of key findings

This study identified two distinct latent profiles among burn patients based on digital health literacy and perceived stigma: a low digital health literacy–high perceived stigma group (40.2%) and a high digital health literacy–low perceived stigma group (59.8%). These findings indicate substantial heterogeneity in how burn survivors access, process, and apply digital health information, as well as how they experience stigma. A significant negative correlation between digital health literacy and perceived stigma suggests that patients with stronger digital competencies tend to experience lower levels of stigma. Social support was found to be an important protective correlate, while higher appearance anxiety and lower education levels were associated with membership in the less adaptive subgroup. Together, these results highlight the intertwined roles of digital literacy, social context, and psychological attributes in influencing the psychosocial recovery of burn patients.

### 4.2 Interpretation of results in light of existing literature

The identification of distinct patient profiles supports the notion that digital health literacy is unevenly distributed among burn survivors, extending previous research beyond variable-centered analyses. Prior studies have shown that higher digital health literacy helps individuals form more realistic expectations and reduces psychosocial distress across chronic diseases (12, 21). In the current study, individuals with high digital literacy appeared better equipped to filter unreliable online information and engage with credible rehabilitation resources, potentially alleviating misconceptions and internalized stigma. This pattern reinforces the theoretical premise that digital literacy is a protective psychosocial resource.

Consistent with social cognitive theory (23), social support emerged as a major correlate influencing this relationship. Sufficient emotional and informational support appears to enhance patients’ self-efficacy in managing digital resources and interpreting online information. Prior evidence suggests that strong family and peer networks help patients regulate emotions, share reliable health knowledge, and feel less isolated (28, 49). Although our study cannot confirm causality, the observed correlation implies that social support and digital health literacy likely reinforce each other, jointly reducing perceived stigma.

Appearance anxiety was another determinant linked to the less adaptive subgroup. Patients highly concerned about their physical appearance may perceive more rejection and tend to avoid online participation or social disclosure, ultimately increasing stigma vulnerability (11). This finding is consistent with studies indicating that high appearance concern reduces psychological flexibility and limits engagement with recovery-oriented information. Thus, interventions addressing appearance anxiety may indirectly strengthen patients’ capacity to benefit from digital health resources.

### 4.3 Practical and clinical implications

These findings provide important practical and clinical insights. First, improving digital health literacy could serve as a cost-effective strategy in burn rehabilitation. Incorporating digital literacy education into post-burn care may empower patients to identify trustworthy online resources, critically evaluate medical information, and protect personal privacy. Hospitals and rehabilitation centers can develop mobile health applications or web-based platforms specifically for burn survivors to deliver tailored education, self-management tools, and peer interaction opportunities.

Second, strengthening social support remains key to reducing perceived stigma. Family-based education, peer mentorship programs, and moderated online support groups may facilitate emotional connection and enhance patients’ confidence in using digital platforms. Clinicians should encourage the involvement of close social networks throughout recovery to reinforce support availability both offline and online.

Third, integrating psychological interventions focused on body image concerns—such as cognitive behavioral therapy or mindfulness-based training—may alleviate appearance anxiety and promote positive engagement with rehabilitation and digital health content. Interventions should be adjusted to patients’ educational backgrounds to ensure inclusiveness, using simplified language or visual-graphic materials where appropriate.

### 4.4 Limitations and future research directions

Several limitations should be acknowledged. First, participants were recruited from hospitals within Sichuan Province, which may restrict the generalizability of findings. Multi-site studies would improve external validity. Second, the cross-sectional design limits causal inference. While digital literacy, social support, and stigma are interrelated, longitudinal or experimental approaches are necessary to clarify temporal sequences and underlying mechanisms.

Third, all data were self-reported, which may be affected by social desirability or recall bias, especially for sensitive constructs like stigma and appearance anxiety. Future work should combine self-reports with behavioral data, such as real digital engagement metrics or clinician evaluations. Finally, the measurement of digital health literacy in this study primarily reflected functional skills; future instruments should encompass competencies relevant to algorithmic transparency and AI-generated health content. Further research is also encouraged to develop and test intervention programs designed for low-literacy, high-stigma groups to evaluate their efficacy in improving psychosocial adaptation and overall wellbeing.

## 5 Conclusion

This study adopted a person-centered approach to elucidate the heterogeneity in digital health literacy and perceived stigma among burn patients, successfully identifying two distinct subgroups: “low digital health literacy–high perceived stigma” and “high digital health literacy–low perceived stigma.” This clear classification underscores significant within-group differences within the burn population, moving beyond traditional variable-centered analyses to offer a more nuanced understanding of how these critical psychosocial factors interact at the individual level. The strong negative correlation between digital health literacy and perceived stigma further highlights the intricate relationship between one’s ability to effectively navigate digital health information and the experience of stigma. Importantly, the analysis pinpointed key factors associated with subgroup membership, with higher levels of social support emerging as a significant protective factor, while elevated appearance anxiety markedly increased the likelihood of being classified within the more vulnerable subgroup. By integrating concepts from social cognitive theory into the digital health domain, this study emphasized the dynamic interplay among individual capabilities, social environments, and psychological states as central to psychosocial adaptation in burn patients.

Practically, results suggest integrating digital health literacy assessments into burn care protocols, with targeted interventions like digital literacy workshops using mobile apps for evidence-based resources and peer support. Enhancing social support through family-integrated programs and addressing appearance anxiety via cognitive-behavioral therapy could associate with shifts toward adaptive profiles, promoting resilience and informed decision-making. Policymakers should prioritize equitable digital infrastructure to mitigate educational disparities, aligning with precision medicine for improved outcomes.

Future research should employ longitudinal designs to explore directionality in digital health literacy-perceived stigma associations and multicenter studies for broader generalizability. Incorporating objective measures (e.g., digital usage data) and additional variables (e.g., resilience, cultural influences) could refine models. Developing and evaluating profile-specific interventions will translate findings into effective strategies, advancing psychosocial care in burns.

## Data Availability

The raw data supporting the conclusions of this article will be made available by the authors, without undue reservation.

## Footnotes

1
https://www.credamo.com/
